# Supplementary material for: The folding propensity of α/sulfono-γ-AA peptidic foldamers with both left- and right-handedness
Source: Commun Chem. 2021 May 10;4:58. doi: 10.1038/s42004-021-00496-0 (PMC9814141; doi:10.1038/s42004-021-00496-0)
Supplement: Supplementary file 1 — Supplementary Information [file 42004_2021_496_MOESM1_ESM.pdf]

Supplementary Information for

The Folding Propensity of α/Sulfono-γ-AA Peptidic Foldamers with Both Left- and Right-Handedness

Peng Teng,[1,2,#,*] Mengmeng Zheng,[1,#] Darrell Cole Cerrato,[1] Yan Shi,[1] Mi Zhou,[1] Songyi Xue,[1] Wei Jiang,[1,3] Lukasz Wojtas,[1] Li-June Ming,[1] Yong Hu, [3,*] and Jianfeng Cai[1,*]

[1]Department of Chemistry, University of South Florida, 4202 E. Fowler Ave, Tampa, FL 33620, USA

[2]Institute of Drug Discovery and Design, College of Pharmaceutical Sciences, Zhejiang University, Hangzhou, Zhejiang 310058, P. R. China

[3]College of Engineering and Applied Science, Nanjing University, Nanjing, Jiangsu 210093, P. R. China

[#]These authors contributed equally.

Correspondence and requests for materials should be addressed to J. C. (jianfengcai@usf.edu), Y. H. (hvyong@nju.edu.cn), or P. T. (pengteng@zju.edu.cn)

1. Supplementary Methods

All reagents and solvents were purchased from Fisher or Aldrich and used without further purification. Fmoc protected *α*-amino acids and Rink-amide resin (0.6 mmol/g, 200–400 mesh) were purchased from Chem-Impex International, Inc. Solid-phase syntheses of the compounds were carried out in the peptide synthesis vessel on a Burrell Wrist-Action shaker. The products were analyzed and purified on a Waters Breeze 2 HPLC system installed with both analytic module (1 mL/min) and preparative module (16 mL/min), by employing a method using 5–100% linear gradient of solvent B (0.1% TFA in acetonitrile) in solvent A (0.1% TFA in water) over 40 min, followed by 100% solvent B over 10 min. The desired fractions were collected and lyophilized on a Labconco lyophilizer. High-resolution MS was conducted on an Agilent 6540 LC/QTOF system. ^{1}HNMR spectra were acquired with the WET solvent suppression at Varian 600 MHz.

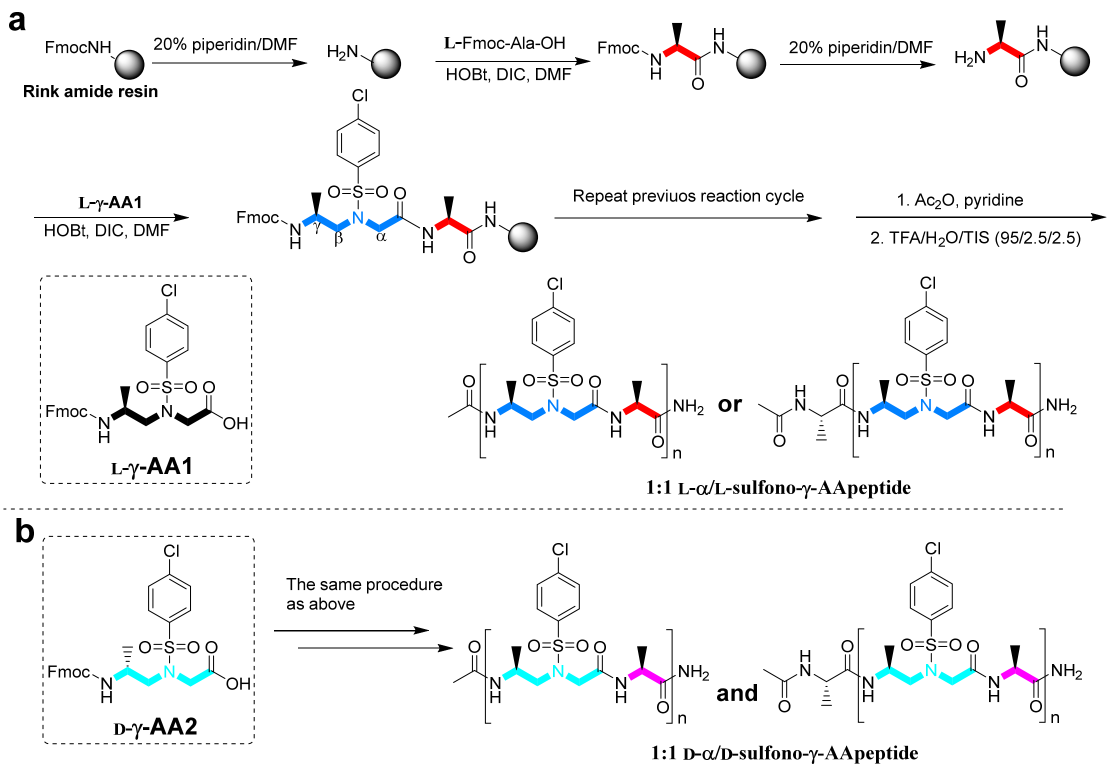

Supplementary Scheme S1. **General synthetic route**. (a) Solid phase to prepare L-α/L-sulfono-γ-AA oligomers; (b) Solid phase to prepare D-α/D-sulfono-γ-AA oligomers.

The L-sulfono-γ-AApeptide building block L-**γ-AA1** was synthesized as previously reported.[1] The D-sulfono-γ-AApeptide building block D-**γ-AA2** was synthesized using same procedures except that the D-Fmoc-Ala-OH was used at the beginning.[2] Oligomers **1–10** were synthesized on solid support Rink-amide resin, as shown in previously reported standard procedures.[1-3] 100 mg of Rink-amide resin were used for the synthesis of each oligomer.

General synthetic procedure of solid phase synthesis of oligomers. The solid phase synthesis was conducted on 100 mg Rink amide resin (0.6 mmol/g) for each oligomer under room temperature at atmosphere pressure. The resin was swelled in DMF for 30 min before use, followed by treatment with 20% piperidine/DMF solution (2 mL) for 10 min (×2) to remove Fmoc protecting group, then washed three times with DCM and three times with DMF. A premixed solution of Fmoc-Ala-OH (with desired chiral configuration**,** 3 equiv.), HOBt (6 equiv.), and DIC (6 equiv.) in 2 mL DMF was added to the resin and shaken for 4 h to complete the coupling reaction. After wash with DCM and DMF, the resin was treated with 20% piperidine/DMF solution for 10 min (×2). Then, the sulfono-γ-AApeptide building block **γ-AA** with the desired chiral configuration was coupled on the resin under the same abovementioned reaction conditions. The reaction cycles were repeated until the desired oligomers were synthesized. The N-terminal of the sequence was capped with acetic anhydride (0.5 mL) in pyridine (2 mL) (30 min×2), followed by treatment with TFA/H_2O/TIS (4 mL, 95/2.5/2.5, v/v/v) for 2 h. The cleavage solution was collected, and the beads was washed with TFA (1 mL×2) and DCM (3 mL×2), the combined organic phase was evaporated under nitrogen flow to give the crude, which was analyzed and then purified by Water HPLC system, with 1 mL/min and 16 mL/min flow speed respectively. The gradient eluting method of 20% to 100% of solvent B (0.1% TFA in acetonitrile) in A (0.1% TFA in water) over 50 min was performed. All the oligomers were obtained with decent crude purity and good yield (55.88–67.04%) after prep-HPLC purification.

Oligomer **1**, HR-MS (ESI), $C_{58}H_{78}Cl_4N_{13}O_{17}S_4$ $[M+H]^+$ calcd = 1496.3270; found = 1496.3268.

Oligomer **2**, HR-MS (ESI), $C_{61}H_{83}Cl_4N_{14}O_{18}S_4$ $[M+H]^+$ calcd = 1567.3641; found = 1567.3637.

Oligomer **3**, HR-MS (ESI), $C_{72}H_{96}Cl_5N_{16}O_{21}S_5$ $[M+H]^+$ calcd = 1855.3977; found = 1855.3951.

Oligomer **4**, HR-MS (ESI), $C_{75}H_{101}Cl_5N_{17}O_{22}S_5$ $[M+H]^+$ calcd = 1926.4348; found = 1926.4308.

Oligomer **5**, HR-MS (ESI), $C_{86}H_{114}C_{l6}N_{19}O_{25}S_6$ $[M+H]^+$ calcd = 2214.4683; found = 2214.4624.

Oligomer **6**, HR-MS (ESI), $C_{89}H_{119}Cl_6N_{20}O_{26}S_6$ $[M+H]^+$ calcd = 2285.5054; found = 1145.2409 $[M+2H]^{2+}$, 1167.2321 $[M+2Na]^{2+}$.

Oligomer **7**, HR-MS (ESI), $C_{100}H_{132}Cl_7N_{22}O_{29}S_7$ $[M+Na]^+$ calcd = 2595.5209; found = 2595.5181.

Oligomer **8**, HR-MS (ESI), $C_{103}H_{137}Cl_7N_{23}O_{30}S_7$ $[M+H]^+$ calcd = 2644.5761; found = 1322.7897 $[M+2H]^{2+}$, 1344.7775 $[M+2Na]^{2+}$.

Oligomer **9**, HR-MS (ESI), $C_{103}H_{136}Cl_7N_{23}O_{30}S_7$ $[M+H]^+$ calcd = 2643.5688; found = 1322.7923 $[M+2H]^{2+}$, 1345.7630 $[M+2Na]^{2+}$, 883.5293 $[M+3H]^{3+}$.

Oligomer **10**, HR-MS (ESI), $C_{117}H_{154}Cl_8N_{26}O_{34}S_8$ $[M+H]^+$ calcd = 3002.6395; found = 1504.8253 $[M+2H]^{2+}$, 1525.7979 $[M+2Na]^{2+}$, 1003.8857 $[M+3H]^{3+}$.

Supplementary Table S1. HPLC purities and retention time of foldamers **1–10**.

Compound	HPLC purification (%)	Retention Time (min)
**1**	99.99	16.55
**2**	99.80	16.06
**3**	99.32	18.47
**4**	99.90	18.30
**5**	100.0	19.81
**6**	99.25	19.24
**7**	97.30	21.31
**8**	99.10	21.61
**9**	97.94	21.03
**10**	98.02	22.59

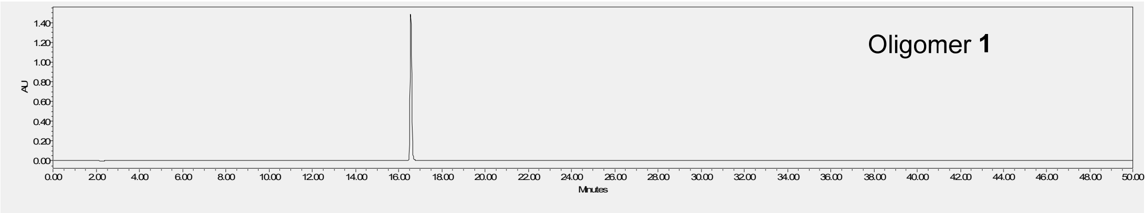
Oligomer 1
AU
Minutes

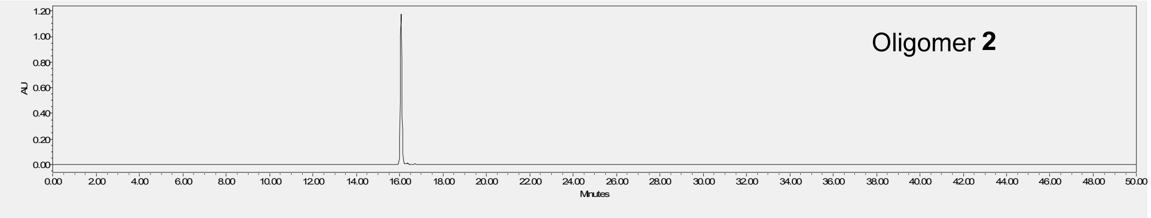
Oligomer 2
AU
Minutes

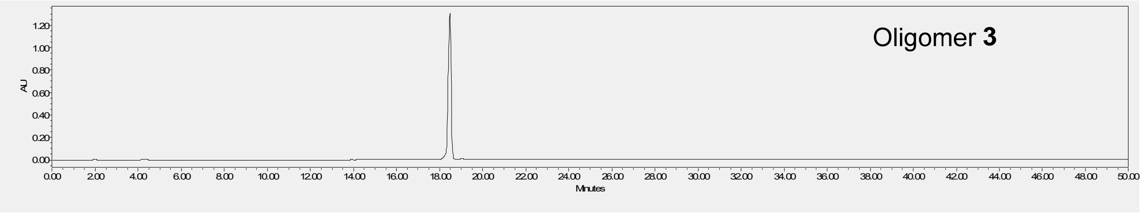
Oligomer 3
AU
Minutes

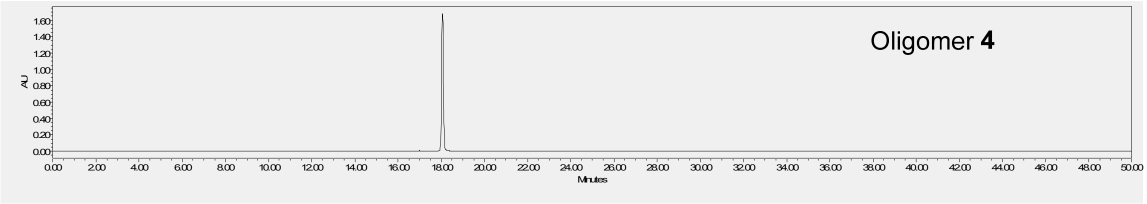
Oligomer 4
AU
Minutes

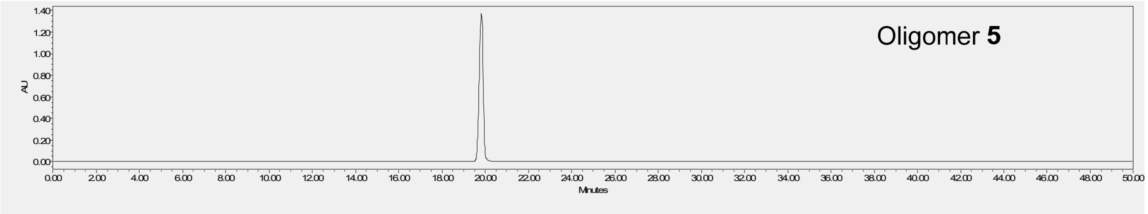
Oligomer 5
AU
Minutes

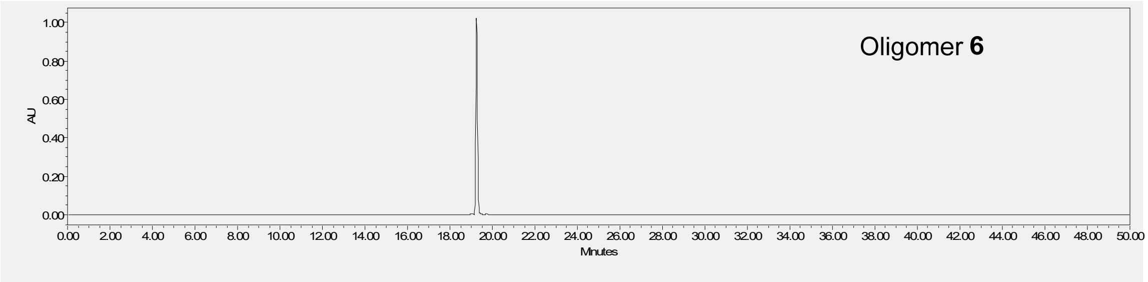
Oligomer 6
AU
Minutes

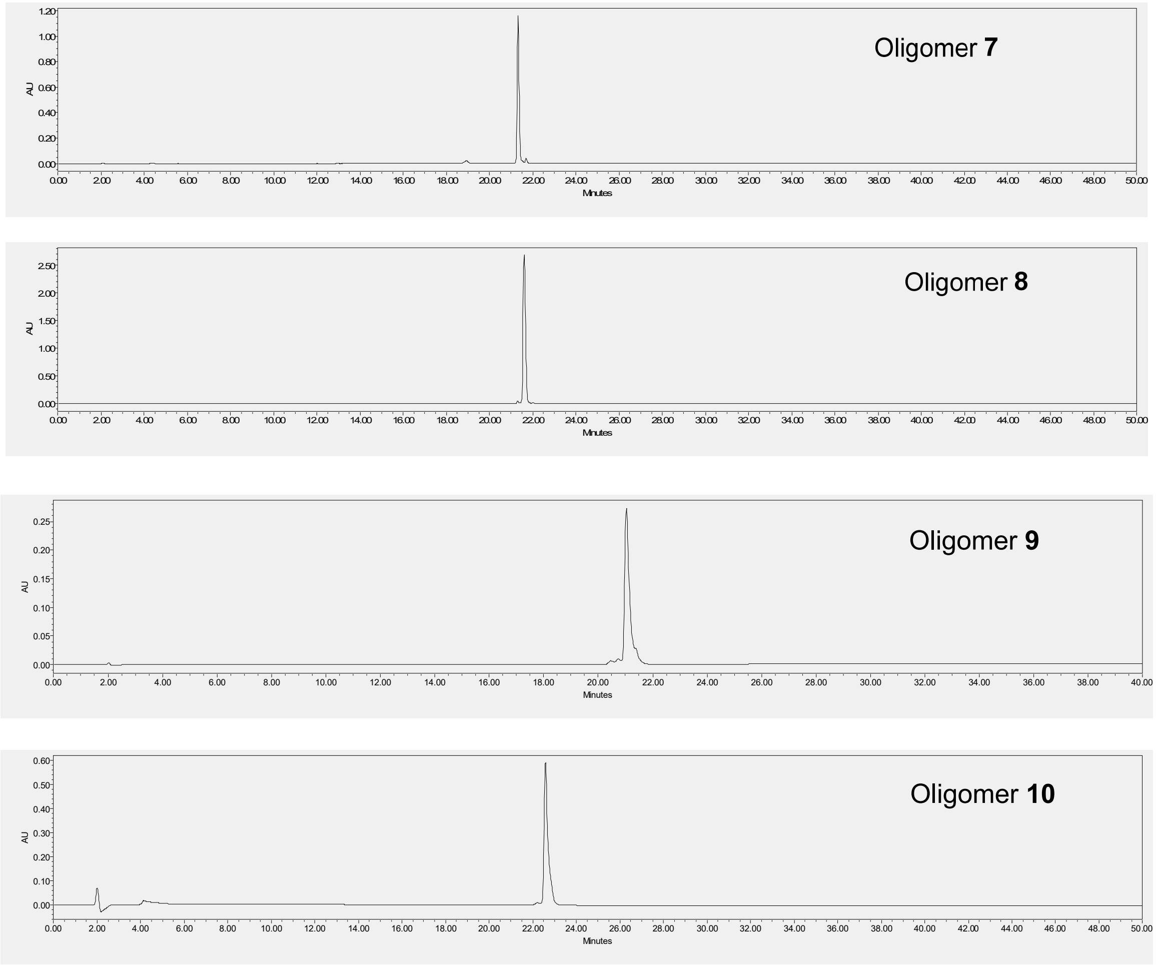

Supplementary Fig. S1. HPLC spectra of foldamers 1–10. The gradient eluting method of 20% to 100% of solvent B (0.1% TFA in acetonitrile) in A (0.1% TFA in water) over 50 min was performed.

Oligomer **1**

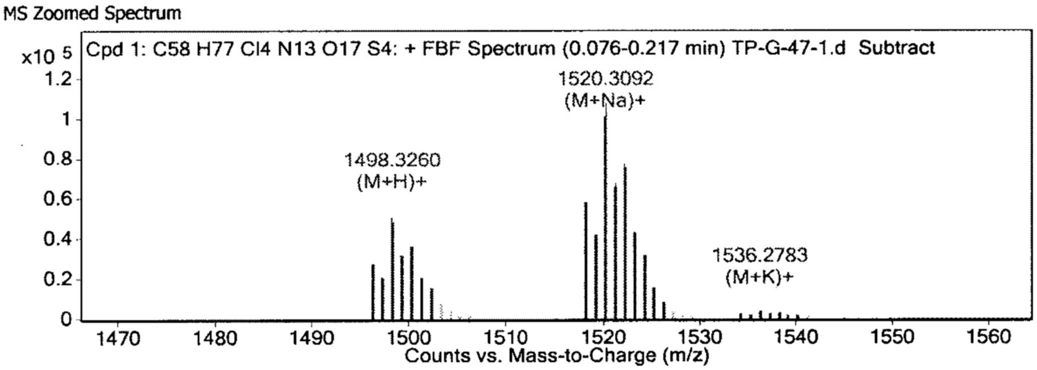

Oligomer **2**

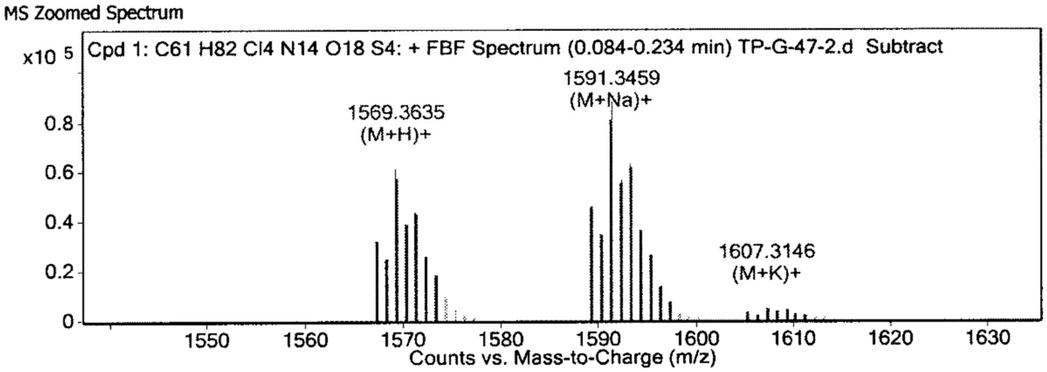

Oligomer **3**

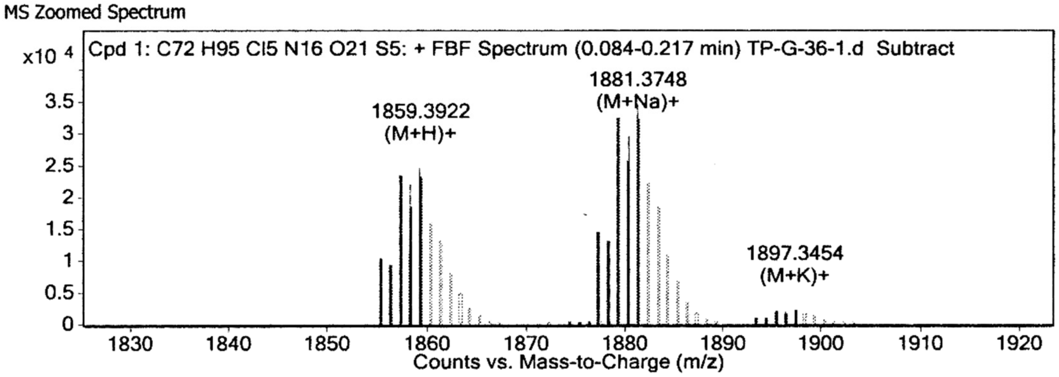

Oligomer **4**

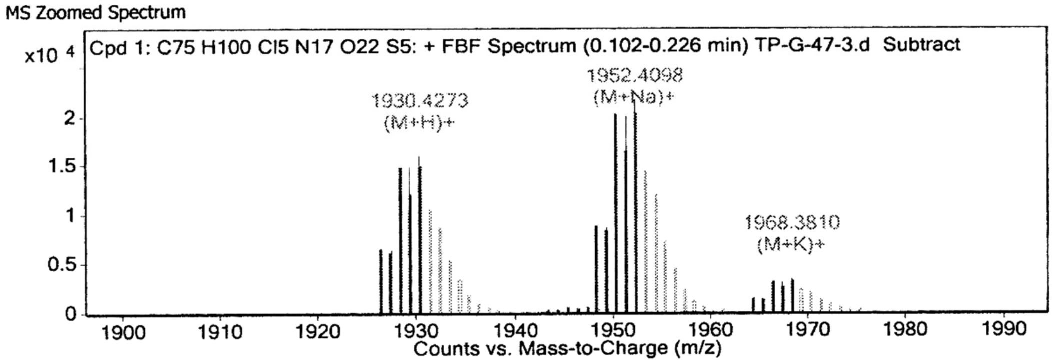

Oligomer **5**

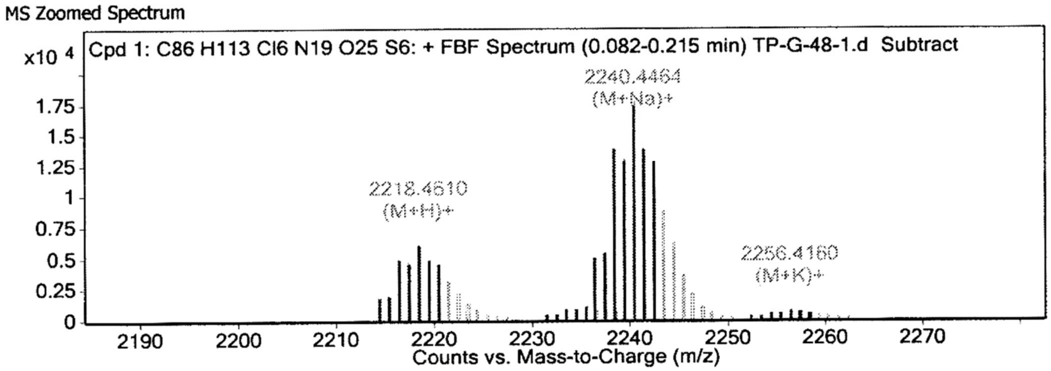

Oligomer **6**

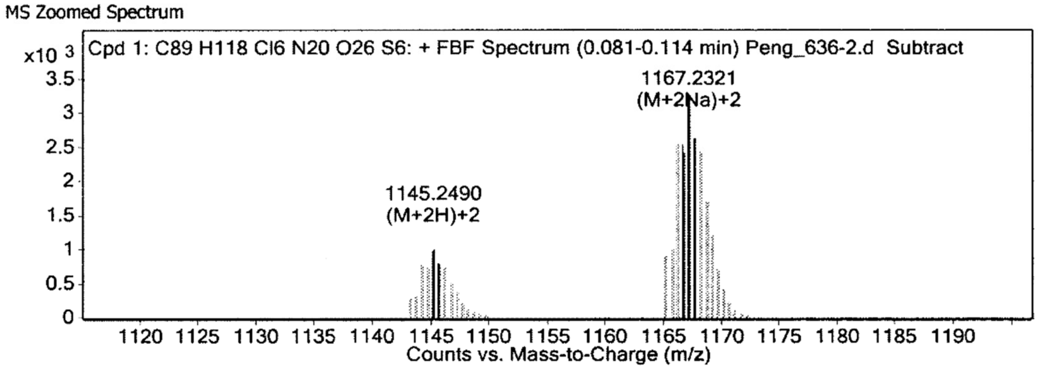

Oligomer 7

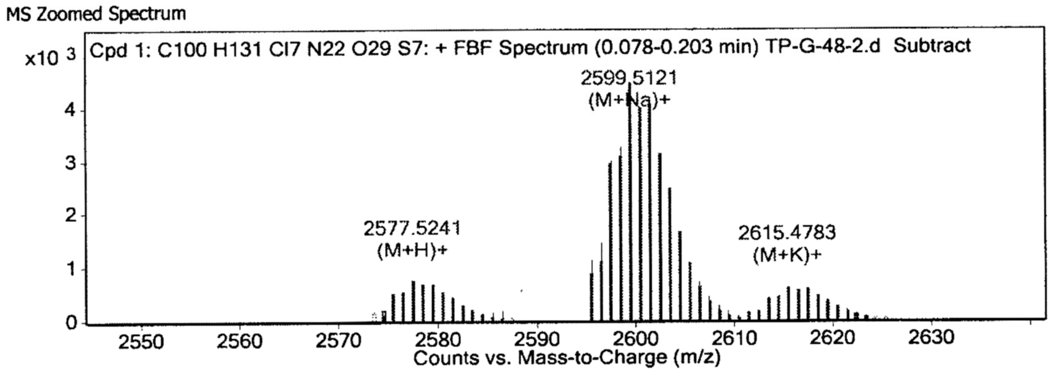

Oligomer 8

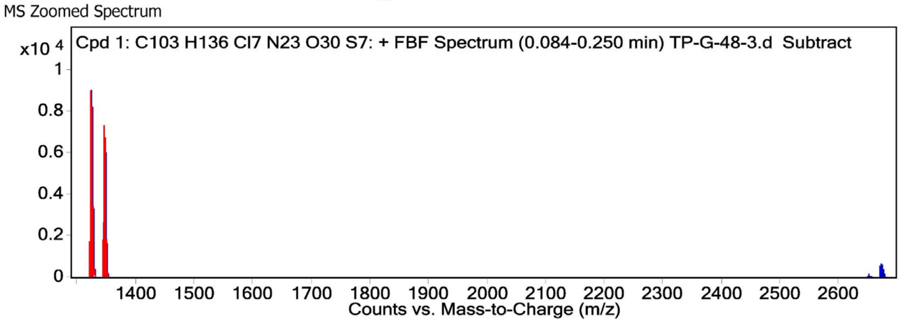

MS Spectrum Peak List

m/z	z	Abund	Formula	Ion
1322.7897	2	1543.92	C103H138Cl7N23O30S7	(M+2H)+2
1324.79	2	9016.68	C103H138Cl7N23O30S7	(M+2H)+2
1325.29	2	8137.86	C103H138Cl7N23O30S7	(M+2H)+2
1325.7888	2	8179.87	C103H138Cl7N23O30S7	(M+2H)+2
1326.289	2	6567.15	C103H138Cl7N23O30S7	(M+2H)+2
1344.7775	2	2405.54	C103H136Cl7N23Na2O30S7	(M+2Na)+2
1346.7719	2	6763	C103H136Cl7N23Na2O30S7	(M+2Na)+2
1347.272	2	6314.27	C103H136Cl7N23Na2O30S7	(M+2Na)+2
2646.5548	1	102.11	C103H137Cl7N23O30S7	(M+H)+
2666.5766	1	367.94	C103H136Cl7N23NaO30S7	(M+Na)+

Oligomer 9

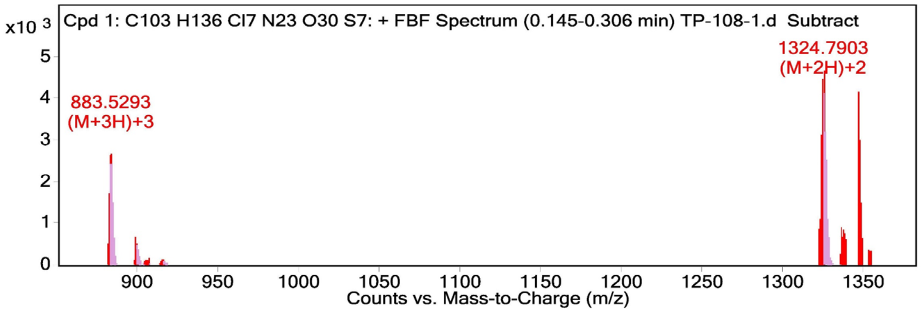

MS Spectrum Peak List

m/z	z	Abund	Formula	Ion
882.1952	3	381.25	C103H139Cl7N23O30S7	(M+3H)+3
898.1774	3	703.99	C103H134Cl7N23Na3O29S7	(M+3Na)+3[-H2O]
904.8322	3	87.29	C103H136Cl7N23Na3O30S7	(M+3Na)+3
914.1518	3	81.25	C103H134Cl7K3N23O29S7	(M+3K)+3[-H2O]
1322.7923	2	608.13	C103H138Cl7N23O30S7	(M+2H)+2
1324.2906	2	3475.52	C103H138Cl7N23O30S7	(M+2H)+2
1324.7903	2	4691.55	C103H138Cl7N23O30S7	(M+2H)+2
1335.78	2	699.87	C103H134Cl7N23Na2O29S7	(M+2Na)+2[-H2O]
1345.763	2	4187.48	C103H136Cl7N23Na2O30S7	(M+2Na)+2
1351.7506	2	368.51	C103H134Cl7K2N23O29S7	(M+2K)+2[-H2O]

Oligomer 10

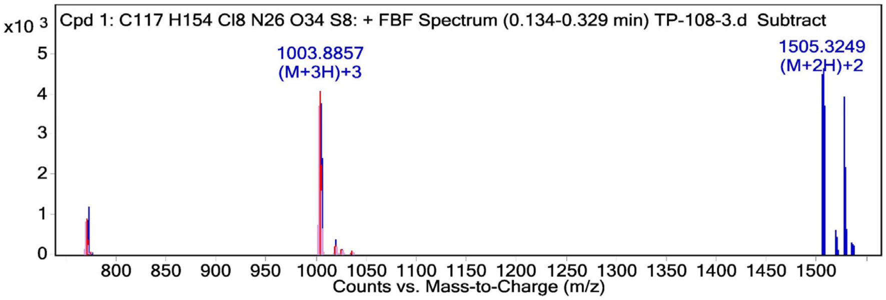

MS Spectrum Peak List

m/z	z	Abund	Formula	Ion
770.6426	4	113.07	C117H152Cl8N26Na4O33S8	(M+4Na)+4[-H2O]
774.8915	4	29.51	C117H154Cl8N26Na4O34S8	(M+4Na)+4
1003.5526	3	3613.13	C117H157Cl8N26O34S8	(M+3H)+3
1018.8664	3	413.37	C117H152Cl8N26Na3O33S8	(M+3Na)+3[-H2O]
1025.1937	3	164.93	C117H154Cl8N26Na3O34S8	(M+3Na)+3
1034.8414	3	89.58	C117H152Cl8K3N26O33S8	(M+3K)+3[-H2O]
1504.8253	2	4518.36	C117H156Cl8N26O34S8	(M+2H)+2
1517.8055	2	633.86	C117H152Cl8N26Na2O33S8	(M+2Na)+2[-H2O]
1525.7979	2	3945.88	C117H154Cl8N26Na2O34S8	(M+2Na)+2
1533.7838	2	328.35	C117H152Cl8K2N26O33S8	(M+2K)+2[-H2O]

Supplementary Fig. S2. HRMS spectra of oligomers 1–10. High-resolution MS was conducted on an Agilent 6540 LC/QTOF system.

Crystal packing of compounds 4, 6, 7, and 9

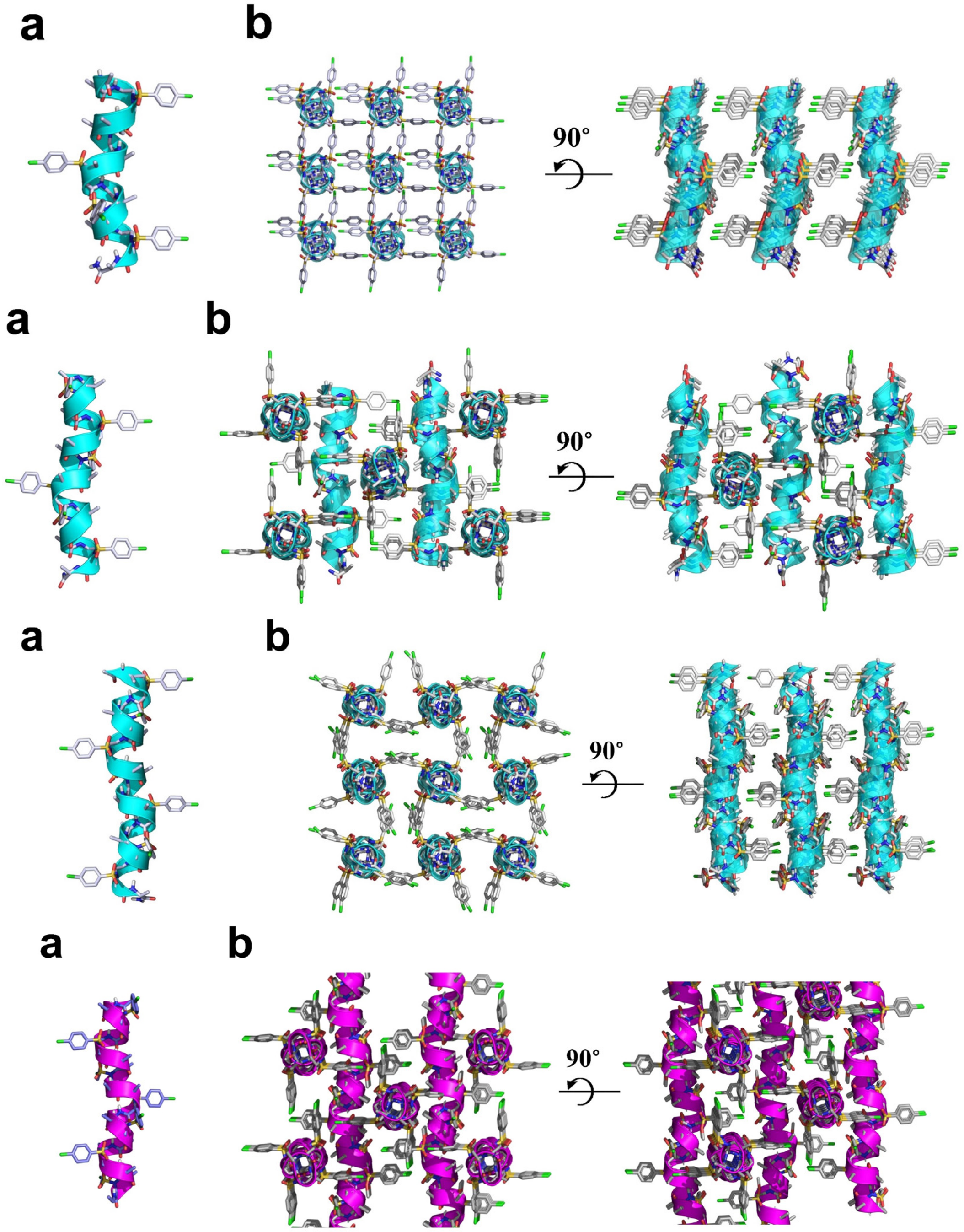

Supplementary Fig. S3. Crystal packing. (a) Helical cartoon representation of **4**, **6**, **7,** or **9** (from top to bottom) shown as dumbbell. (b) Crystal packing of **4**, **6**, **7,** or **9**. Solvent molecules were also excluded from the crystal lattice.

NMR studies of oligomer 8

The NMR spectra were obtained on a Varian VNMRS 600 MHz spectrometer equipped with four RF channels and a Z-axis-pulse-field gradient cold probe. Oligomer **8** was dissolved in 0.5 mL of CD_3OH in a 5 mm NMR tube at a concentration of 4 mM. The ^1H shift assignment was achieved by sequential assignment procedures based on DQFCOSY, COSY, zTOCSY and NOESY measurement. COCSY and NOESY spectra were acquired with the Wet solvent suppression at Varian 600 MHz at 10 °C. All experiments were performed by collecting 4096 points in f2 and 300 points in f1. A DIPSI2 spin lock sequence with a spin lock field of 6k Hz and mixing time of 80 ms were used in zTOCSY. NOESY experiment was carried out using a mix time of 200 ms. Vnmrj was used to process and analyze 2D NMR data.

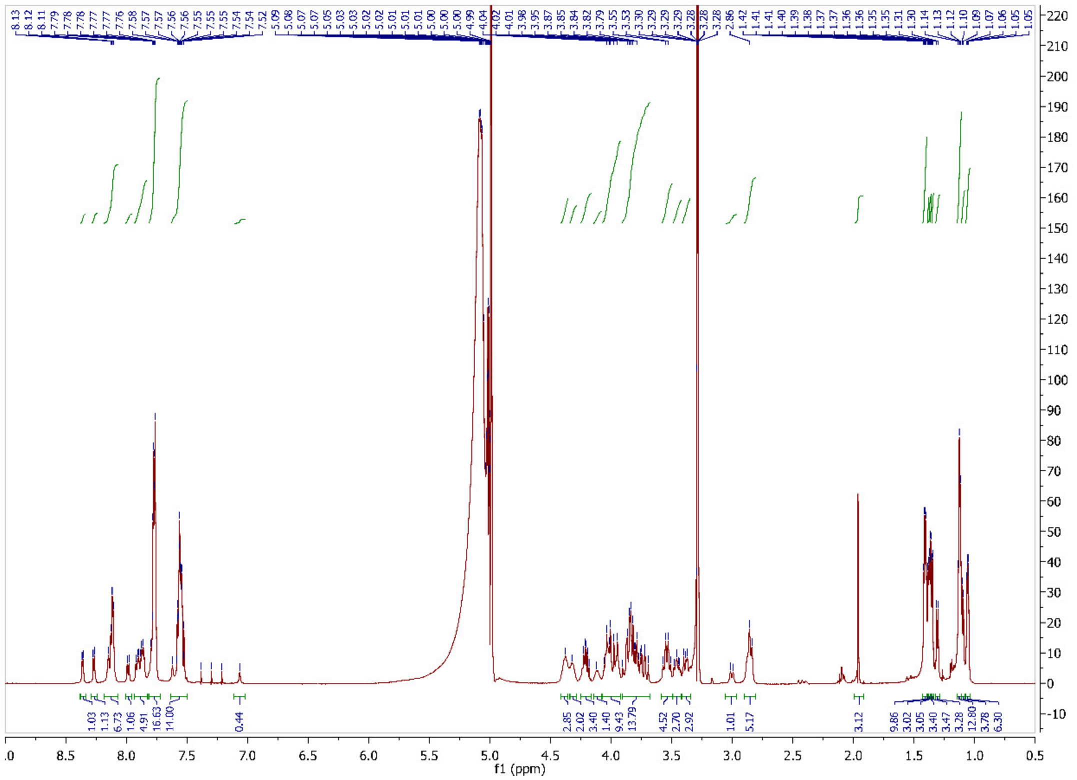

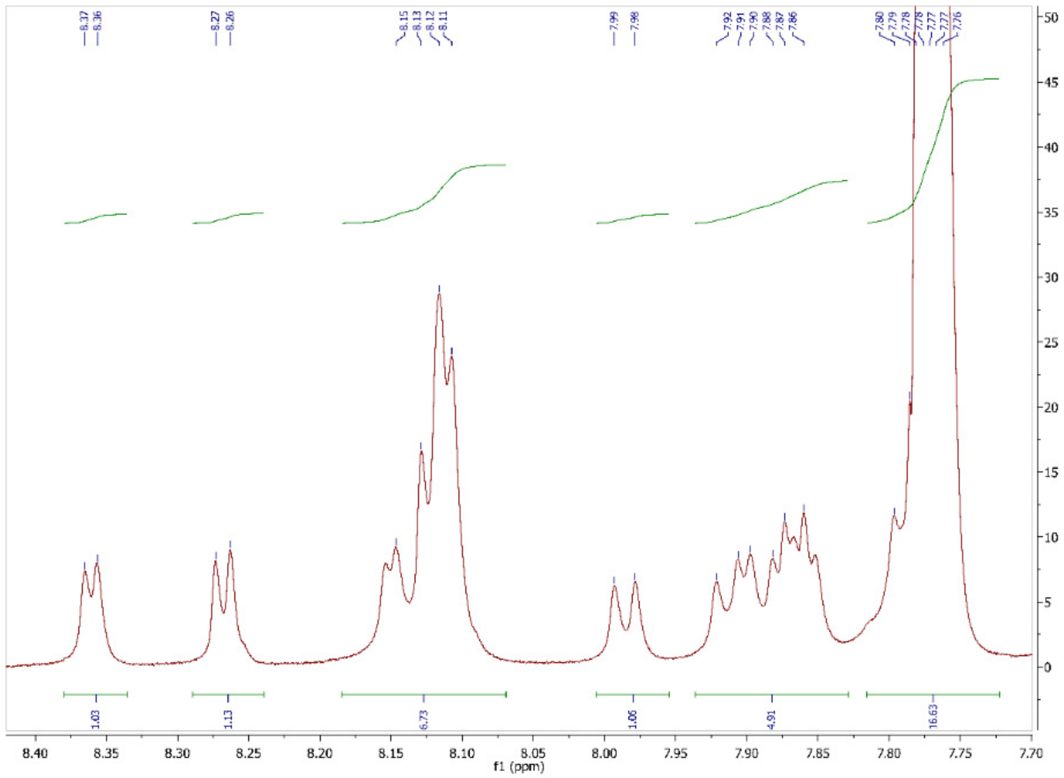

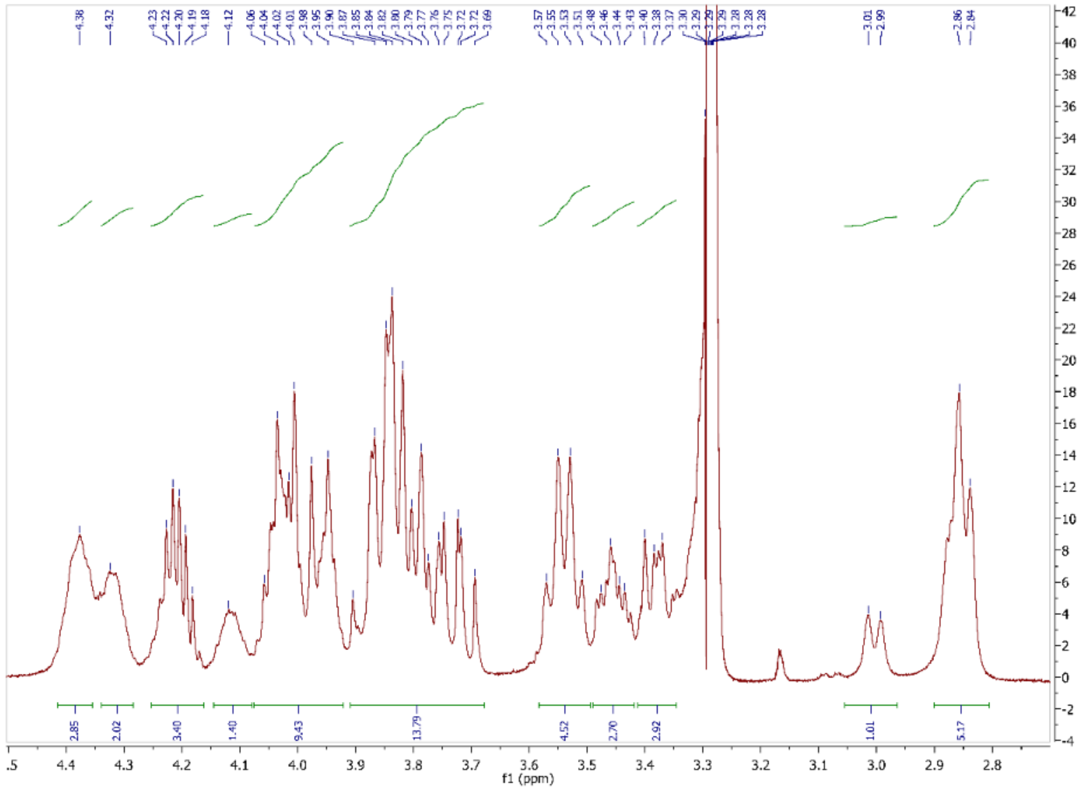

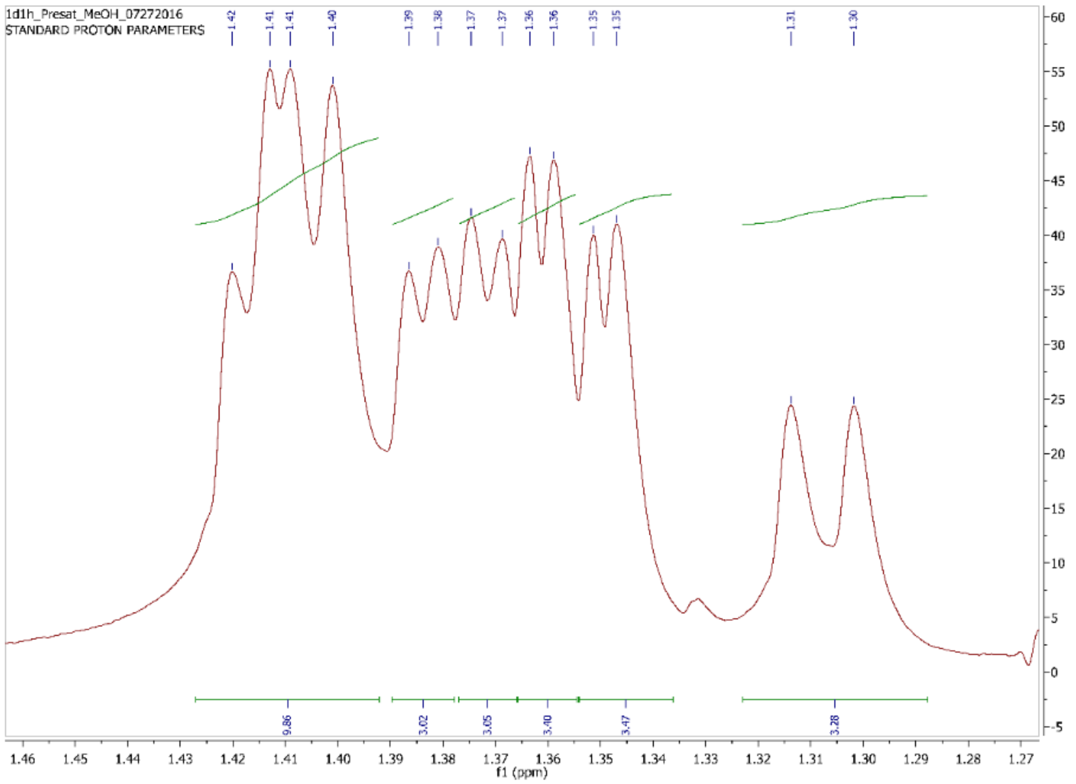

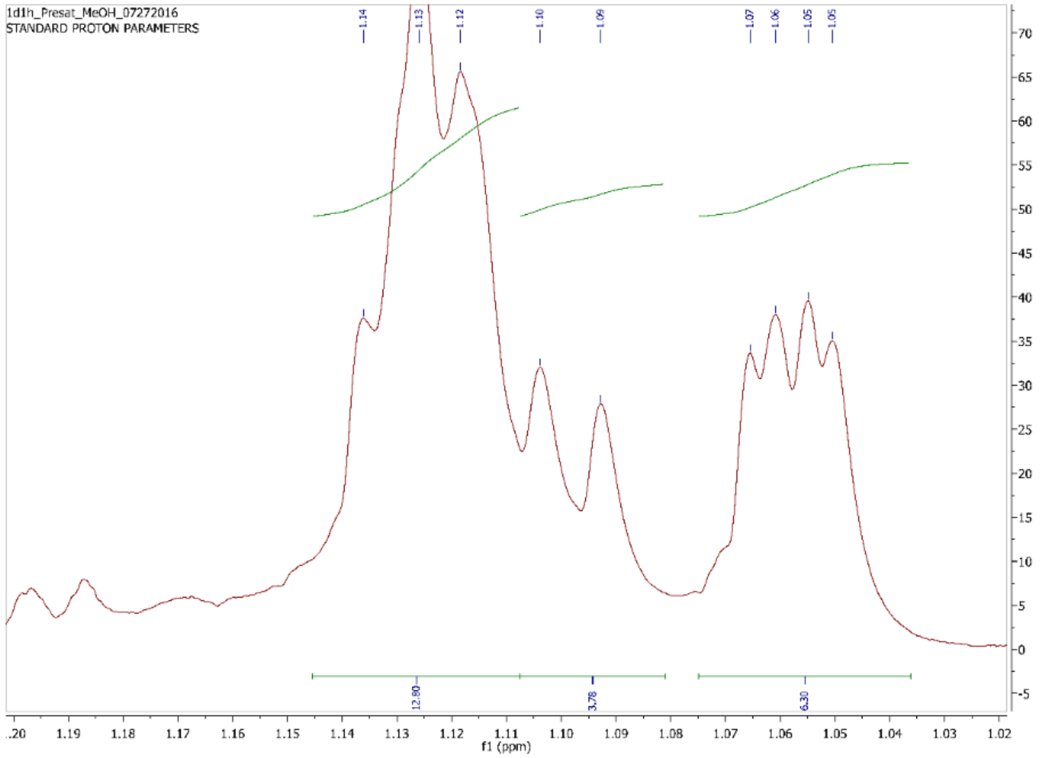

Supplementary Fig. S4. 1D NMR of oligomer 8. ^{1}H NMR spectra of **8** in CD_3OH.

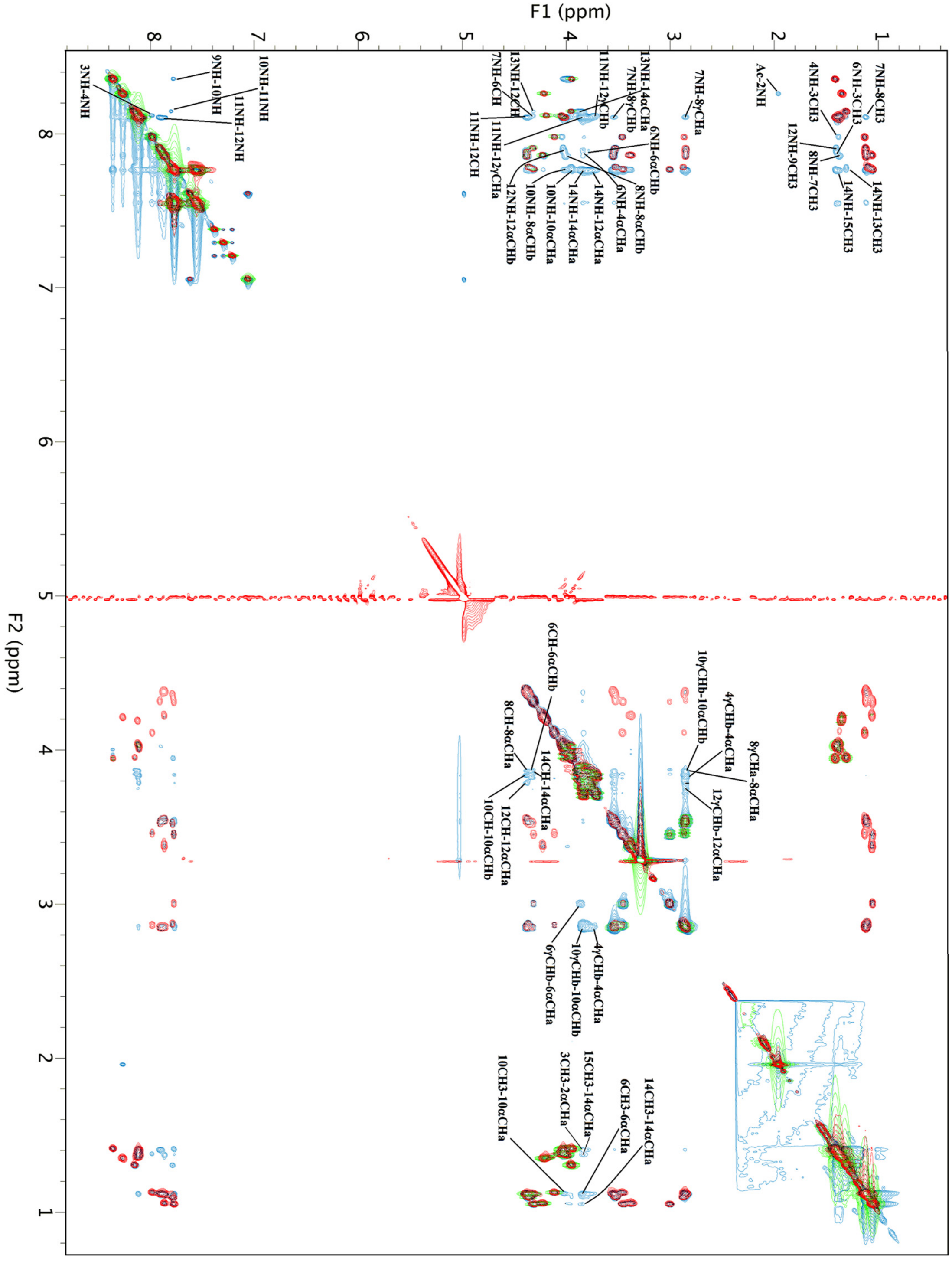
F1 (ppm)
F2 (ppm)
7NH-8CH3
6NH-3CH3
4NH-3CH3
12NH-9CH3
8NH-7CH3
14NH-15CH3
14NH-13CH3
Ac-2NH
7NH-8γCHa
6NH-6αCHb
13NH-14αCHa
7NH-8γCHb
11NH-12γCHb
8NH-8αCHb
6NH-4αCHa
14NH-12αCHa
14NH-14αCHa
10NH-10αCHa
10NH-8αCHb
12NH-12αCHb
11NH-12γCHa
13NH-12CH
7NH-6CH
11NH-12CH
10NH-11NH
9NH-10NH
11NH-12NH
3NH-4NH
10γCHb-10αCHb
4γCHb-4αCHa
8γCHa-8αCHa
12γCHb-12αCHa
6CH-6αCHb
8CH-8αCHa
14CH-14αCHa
12CH-12αCHa
10CH-10αCHb
4γCHb-4αCHa
10γCHb-10αCHb
6γCHb-6αCHa
14CH3-14αCHa
6CH3-6αCHa
15CH3-14αCHa
3CH3-2αCHa
10CH3-10αCHa

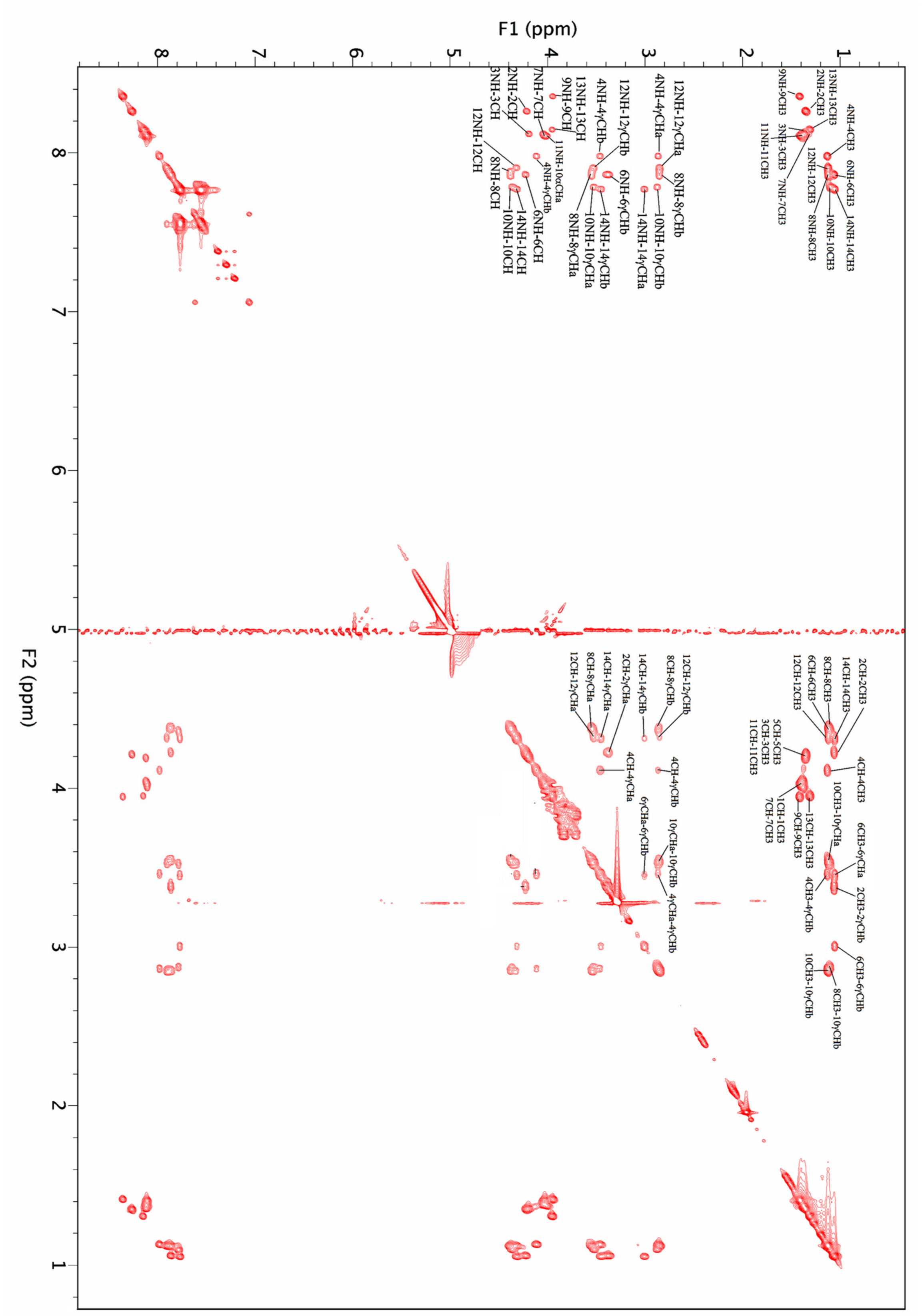
F1 (ppm)
F2 (ppm)

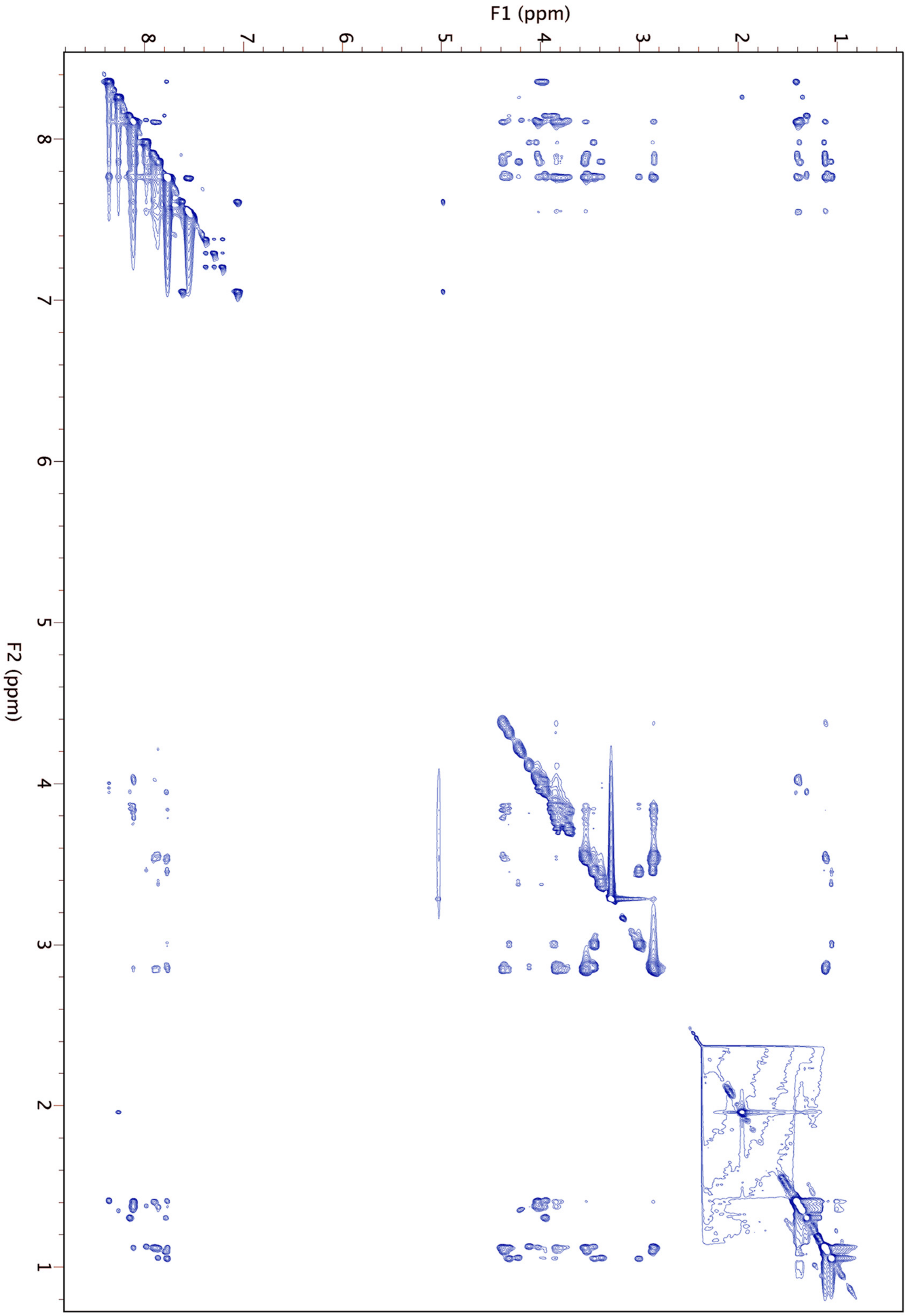
F1 (ppm)
F2 (ppm)

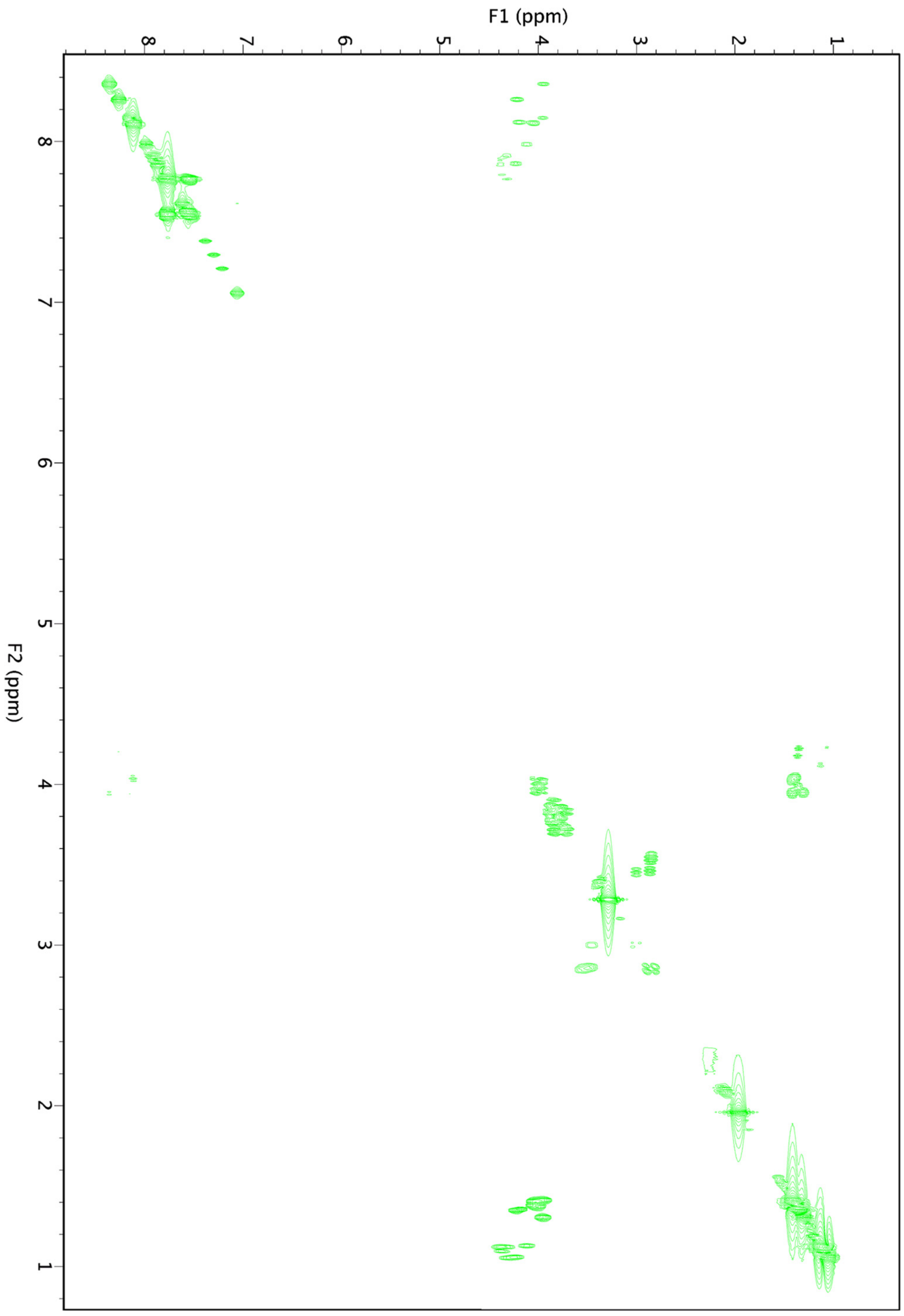

Supplementary Fig. S5. Annotated natural 2D spectra for oligomer 8. The spectra were collected at 600 MHz at a temperature of 10 ºC. Chemical shift assignments were based on ^{1}H, ^{1}H-zTOCSY (red color), ^{1}H-NOESY (blue color), and ^{1}H-COSY (green color).

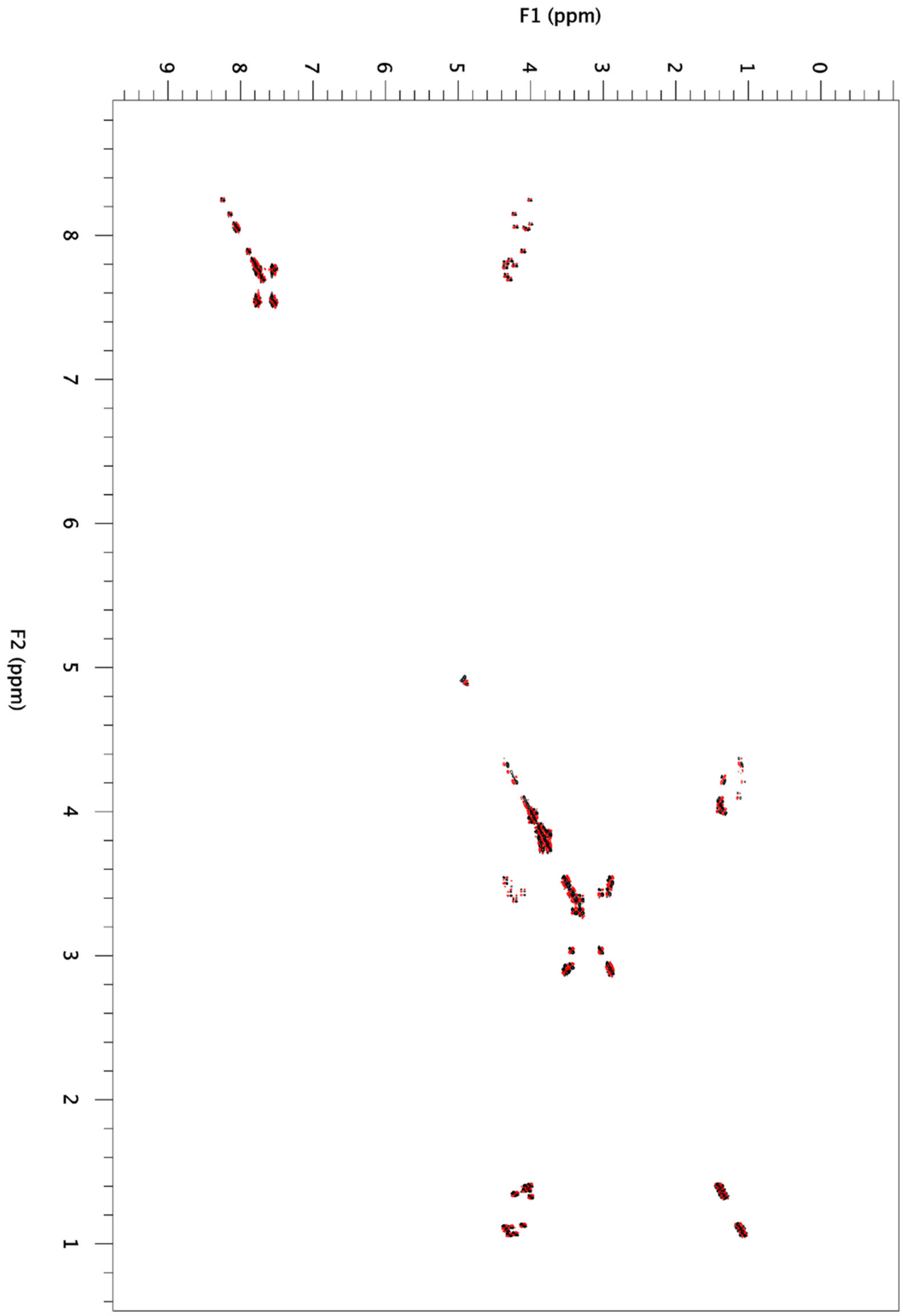

Supplementary Fig. S6. DQFCOSY spectrum for oligomer 8. The spectra were collected at 600 MHz at a temperature of 10 ºC.

2. Supplementary Discussion

Lyophilized powders of oligomers **6** (3 mg), **9** (3 mg) and **10** (2 mg) were dissolved in 2 mL of dichloromethane/acetonitrile (20:80, v/v) and then left for slow evaporation at room temperature within two days to give crystals. Lyophilized powders of oligomer **4** (2 mg) were dissolved in THF (2 mL) and then pentane (1 mL) was diffused slowly into THF layer, crystals were formed in a week. Crystals of **7** were obtained from slow evaporation of 3 mg/mL solution in chloroform. Oligomer **2** was also crystalized from slow diffusion of pentane into THF in ten days, however, the crystals were not of good quality for X-ray diffraction (diffraction up to 5.00 Å of resolution only). Foldamers **8** and **9** in 1:1 ratio (4 mg, the racemate **11**) was crystallized from CH_2Cl_2/CH_3CN (60:40, v/v) using slow vaporization over two days.

Both compounds **4** and **7** crystallize in $P2_1$ space group with one molecule in the asymmetric unit. While compounds **6** and **8** crystallize in $P4_12_12$ space group with two α peptide and two sulfono-γ-AA peptide residues in the asymmetric unit. In contrast, compounds **9** and **10** crystallize in $P4_32_12$ space group with two α peptide and two sulfono-γ-AA peptide residues in the asymmetric unit. The apparent infinite chain in structures **6**, **9**, and **10** is an effect of translational disorder in those structures and occupancy of atoms in model were adjusted to match the appropriate formulas, therefore the N-terminal acetyl group was not visible.

The X-ray diffraction data for all compounds were measured on Bruker D8 Venture PHOTON 100 CMOS system equipped with a Cu K_α INCOATEC Imus micro-focus source (λ = 1.54178 Å). Indexing was performed using APEX3[4] (Difference Vectors method). Data integration and reduction were performed using SaintPlus 6.01[5]. Absorption correction was performed by multi-scan method implemented in SADABS[6]. Space groups were determined using XPREP implemented in APEX3. Structures were solved using SHELXT or SHELXD and refined using SHELXL-2014[7-9] (full-matrix least-squares on F^2) through OLEX2 interface program[10].

For compound **4**, diffraction spots were observed up to ca. 1.1 Å resolution. The structure has been solved using program Shelxd[11] and refined using geometry and ADP restraints. Inspection

of 2Fo-Fc electron density map in WinCoot[12] suggested the THF as disordered solvent. THF was subsequently modeled into the map, real space refined with WinCoot and finally refined with Shelxl with fixed occupancy and restraints. The group of electron density peaks located between aromatic rings of two helices have been tentatively assigned as pyridine and refined with restrains. Crystal data and refinement conditions are shown in Table S3.

Supplementary Table S2: Crystal data and structure refinement for Oligomer **4**.

Identification code	PT_G47_3_0422
Empirical formula	$C_{102}H_{149}Cl_5N_{18}O_{27.5}S_5$
Empirical formula	$C_{75}H_{100}Cl_5N_{17}O_{22}S_5 \cdot 5.5THF \cdot C_5H_5N$
Formula weight	2404.93
Temperature/K	100(2)
Crystal system	monoclinic
Space group	$P2_1$
a/Å	12.5708(14)
b/Å	51.249(6)
c/Å	12.9887(14)
α/°	90
β/°	90.116(2)
γ/°	90
Volume/Å^3	8367.8(16)
Z	2
ρ_{calc}g/cm^3	0.954
μ/mm^{-1}	1.835
F(000)	2544.0
Crystal size/mm^3	0.400 × 0.090 × 0.080
Radiation	CuKα (λ = 1.54178)
2Θ range for data collection/°	6.806 to 89.062
Index ranges	$-11 \le h \le 11$, $-46 \le k \le 46$, $-11 \le l \le 11$
Reflections collected	55363
Independent reflections	12960 [R_{int} = 0.0859, R_{sigma} = 0.1006]
Data/restraints/parameters	12960/2258/1403
Goodness-of-fit on F^2	1.254
Final R indexes [I>=2σ (I)]	R_1 = 0.1308, wR_2 = 0.3169
Final R indexes [all data]	R_1 = 0.1839, wR_2 = 0.3451
Largest diff. peak/hole / e Å^{-3}	0.55/-0.49
Flack parameter	0.124(8)

For compound **6**, the structure solution has led to apparent "infinite" helix which can be explained through the presence of translational disorder between discrete chains in the crystal. The occupancy of each of two C3NSO2PhCl parts (of sulfono-γ-AApeptide) has been adjusted to 0.857 in asymmetric unit to match the ratio of alanine to sulfono-γ-AApeptide (7:6). From the disorder point of view when C3NSO2PhCl part is missing the terminal part of peptide is present leading to the column formed out of peptide helices interacting through hydrogen bonds. The disordered Ph-Cl groups and solvent (CH_3CN) have been refined using restraints. Crystal data and refinement conditions are shown in Table S4.

Supplementary Table S3: Crystal data and structure refinement for oligomer **6.**

Identification code	pt_g_36_2_0m
Empirical formula	$C_{106.5}H_{144.25}Cl_6N_{28.75}O_{26}S_6$
Moiety Formula	$C_{89}H_{118}Cl_6N_{20}O_{26}S_6$, 8.75$CH_3CN$
Formula weight	2648.18
Temperature/K	100
Crystal system	tetragonal
Space group	$P4_12_12$
a/Å	17.1537(4)
b/Å	17.1537(4)
c/Å	28.9825(8)
α/°	90
β/°	90
γ/°	90
Volume/Å^3	8528.1(5)
Z	2.28571
ρ_{calc}g/cm^3	1.179
μ/mm^{-1}	2.406
F(000)	3178.0
Crystal size/mm^3	0.140 × 0.140 × 0.120
Radiation	CuKα (λ = 1.54178)
2Θ range for data collection/°	7.288 to 138.224
Index ranges	$-20 \leq h \leq 20$, $-20 \leq k \leq 20$, $-34 \leq l \leq 32$
Reflections collected	64938
Independent reflections	7920 [R_{int} = 0.0514, R_{sigma} = 0.0337]
Data/restraints/parameters	7920/244/503

Goodness-of-fit on F^2	1.057
Final R indexes [I>=2σ (I)]	R_1 = 0.0985, wR_2 = 0.2669
Final R indexes [all data]	R_1 = 0.1304, wR_2 = 0.3149
Largest diff. peak/hole / e Å^{-3}	0.55/-0.40
Flack parameter	0.041(10)

For compound **7**, Diffraction spots were observed only up to ca. 1.2 – 1.30 Å resolution. The structure has been solved using program Shelxd [11] using NSO_2 fragment seeding and completed using Fourier methods in Shelxl. Due to low resolution and disorder, restraints were necessary to refine positions and anisotropic thermal parameters. Crystal was refined as pseudo-merohedral four component twin with 0 1 0 -1 0 0 0 0 1 4 twin law. After each refinement run the 2Fo-Fc map has been inspected using Program WinCoot, especially for significantly disordered terminal residues, to assure that atoms are located within the map contours (at 0.6–1 sigma level). Distances of O…N hydrogen bonds were restrained at terminal sides to prevent drifting of disordered residues. Disordered chloroform and tentative MeCN/MeOH molecules were refined using restraints. The inspection of 2Fo-Fc electron density map in WinCoot had clearly suggested the $CHCl_3$ as disordered solvent. $CHCl_3$ was subsequently modeled into the map, real space refined with WinCoot and finally refined with Shelxl with fixed occupancy and restraints. Some of the larger residual electron density peaks have been tentatively assigned as Cl with assumption they are a part of heavily disordered chloroform molecules that could not be easily localized. The relatively high R-factor is caused mostly by unaccounted disordered solvent molecules – it can be lowered to ~11% through application of Squeeze solvent mask procedure. This however eliminates chloroform molecules which are important part of crystal packing so solvent corrected data have not been presented for publication. Although the data quality is low per small molecule crystallography standards the main goal of structural analysis was to establish secondary structure, for which purpose the data quality is sufficient in authors' opinion. The relatively high R-factor is caused mostly by unaccounted disordered solvent molecules, which can be lowered to ~ 10% through

application of Squeeze procedure. This however eliminates solvent molecules which are important part of crystal packing. Two models of crystal structure are provided: 1) including solvent (Table S4) and 2) with solvent mask where disordered solvent molecules and counterions were treated as diffuse using Platon Squeeze procedure (Table S5).[13]

Supplementary Table S4: Crystal data and structure refinement for oligomer **7**_solvent**.**

Identification code	TP48_2_0m
Empirical formula	$C_{114.82}H_{136.32}Cl_{22.95}N_{25}O_{33.5}S_7$
Moiety formula	$C_{100}H_{131}Cl_7N_{22}O_{29}S_7$, 5.32($CHCl_3$), 3($C_2$ N), 3.5(CO), O
Formula weight	3440.57
Temperature/K	100.04
Crystal system	monoclinic
Space group	$P2_1$
a/Å	17.1947(15)
b/Å	17.2126(16)
c/Å	35.063(3)
α/°	90
β/°	90.043(4)
γ/°	90
Volume/Å^3	10377.5(16)
Z	2
ρ_{calc}g/cm^3	1.101
μ/mm^{-1}	3.911
F(000)	3541.0
Crystal size/mm^3	0.120 × 0.100 × 0.060
Radiation	CuKα (λ = 1.54178)
2Θ range for data collection/°	5.134 to 74.162
Index ranges	$-13 \leq h \leq 13$, $-13 \leq k \leq 13$, $-26 \leq l \leq 26$
Reflections collected	23651
Independent reflections	9355 [R_{int} = 0.0691, R_{sigma} = 0.0822]
Data/restraints/parameters	9355/3223/1853
Goodness-of-fit on F^2	1.535
Final R indexes [I>=2σ (I)]	R_1 = 0.1557, wR_2 = 0.3341
Final R indexes [all data]	R_1 = 0.1955, wR_2 = 0.3845
Largest diff. peak/hole / e Å^{-3}	0.56/-0.43
Flack parameter	0.012(16)

Table S5 Crystal data and structure refinement for oligomer **7**_solvent_mask.

Identification code	Oligomer **7**_solvent_mask
Empirical formula	$C_{100}H_{131}Cl_7N_{22}O_{29}S_7$
Formula weight	2577.83
Temperature/K	100.04
Crystal system	monoclinic
Space group	$P2_1$
a/Å	17.1947(15)
b/Å	17.2126(16)
c/Å	35.063(3)
α/°	90
β/°	90.043(4)
γ/°	90
Volume/Å^3	10377.5(16)
Z	2
ρ_{calc}g/cm^3	0.825
μ/mm^{-1}	1.930
F(000)	2696.0
Crystal size/mm^3	0.12 × 0.1 × 0.06
Radiation	CuKα (λ = 1.54178)
2Θ range for data collection/°	5.14 to 71.462
Index ranges	-13 ≤ h ≤ 13, -13 ≤ k ≤ 13, -26 ≤ l ≤ 26
Reflections collected	9342
Independent reflections	9342 [R_{int} = 0.0691, R_{sigma} = 0.0819]
Data/restraints/parameters	9342/2807/1486
Goodness-of-fit on F^2	1.063
Final R indexes [I>=2σ (I)]	R_1 = 0.0973, wR_2 = 0.2443
Final R indexes [all data]	R_1 = 0.1329, wR_2 = 0.2972
Largest diff. peak/hole / e Å^{-3}	0.45/-0.31
Flack parameter	0.29(3)

Oligomers **9**, **10**, and **11** diffracted up to 0.95 Å resolution. Disordered parts were refined using restraints. Data processing and structure solution of all structures have initially led to apparent "infinite" polymeric" helix and structure with unit cell parameters along the axis of helix shorter than the length of actual helix. This could be explained as an effect of translational disorder between

discrete peptide chains in the crystal as already discussed in previous publication.[1] The possible explanation is that discrete peptide chains interact through hydrogen bonds at terminal points to form a “column”. Adjacent “Columns” (helices) are interacting weekly. Because seven (or eight) γ-peptide residues are present in single helix, several packing modes are possible, with adjacent helices translated or translated and rotated so that the same weak stabilizing interactions are still present. This leads to translational disorder and diffraction pattern resembling the one from hypothetical structure with “infinite-like” polymeric peptide chains. In reality, the C3NSO2Ph part of the apparent infinite chain is missing at sites where every 8th (or 9th) γ-peptide residue would reside in hypothetical infinite helix. The gap is where hydrogen bond interactions between terminal parts of polypeptides take place. This is accounted for in the model by lower than one occupancy of corresponding part of γ-peptide. The contribution of disordered content in structural voids was treated as diffuse using Squeeze procedure implemented in Platon program.[14,15] Crystal data and refinement conditions are shown in Tables S6-S8.

Supplementary Table S6. Crystal data and structure refinement for oligomer **9.**

Identification code	JC_TP_H_108_1
Empirical formula	$C_{103}H_{136}Cl_7N_{23}O_{30}S_7$
Formula weight	2648.91
Temperature/K	99.99
Crystal system	tetragonal
Space group	$P4_32_12$
a/Å	17.1371(4)
b/Å	17.1371(4)
c/Å	29.0104(8)
α/°	90
β/°	90
γ/°	90
Volume/Å^3	8519.8(5)
Z	2
ρ_{calc}g/cm^3	1.033
μ/mm^{-1}	2.369
F(000)	2772.0

Crystal size/mm^3	0.33 × 0.15 × 0.13
Radiation	CuKα (λ = 1.54178)
2Θ range for data collection/°	7.294 to 149.614
Index ranges	-20 ≤ h ≤ 21, -19 ≤ k ≤ 16, -34 ≤ l ≤ 36
Reflections collected	74789
Independent reflections	8717 [R_{int} = 0.0492, R_{sigma} = 0.0328]
Data/restraints/parameters	8717/96/419
Goodness-of-fit on F^2	1.069
Final R indexes [I>=2σ (I)]	R_1 = 0.0661, wR_2 = 0.2052
Final R indexes [all data]	R_1 = 0.0755, wR_2 = 0.2183
Largest diff. peak/hole / e Å^{-3}	0.32/-0.29
Flack parameter	0.143(8)

Supplementary Table S7. Crystal data and structure refinement for oligomer **10.**

Identification code	TP_H_108_3
Empirical formula	$C_{117}H_{154}Cl_8N_{26}O_{34}S_8$
Formula weight	3008.62
Temperature/K	99.98
Crystal system	tetragonal
Space group	$P4_32_12$
a/Å	17.1367(3)
b/Å	17.1367(3)
c/Å	28.9848(11)
α/°	90
β/°	90
γ/°	90
Volume/Å^3	8511.9(4)
Z	1.77776
ρ_{calc}g/cm^3	1.043
μ/mm^{-1}	2.404
F(000)	2798.0
Crystal size/mm^3	0.37 × 0.11 × 0.09
Radiation	CuKα (λ = 1.54178)
2Θ range for data collection/°	5.992 to 108.528
Index ranges	-18 ≤ h ≤ 18, -18 ≤ k ≤ 18, -28 ≤ l ≤ 30
Reflections collected	69116
Independent reflections	5205 [R_{int} = 0.1112, R_{sigma} = 0.0373]
Data/restraints/parameters	5205/204/419

Goodness-of-fit on F^2	1.044
Final R indexes [I>=2σ (I)]	R_1 = 0.0645, wR_2 = 0.1718
Final R indexes [all data]	R_1 = 0.1012, wR_2 = 0.2002
Largest diff. peak/hole / e Å^{-3}	0.17/-0.19
Flack parameter	0.045(11)

Supplementary Table S8. Crystal data and structure refinement for oligomer **11.**

Identification code	TP_7_MIX
Empirical formula	$C_{103}H_{136}Cl_7N_{23}O_{30}S_7$
Formula weight	2648.91
Temperature/K	150.0
Crystal system	tetragonal
Space group	$I4_1/a$
a/Å	21.1606(12)
b/Å	21.1606(12)
c/Å	17.5048(11)
α/°	90
β/°	90
γ/°	90
Volume/Å^3	7838.1(10)
Z	2
ρ_{calc}g/cm^3	1.122
μ/mm^{-1}	2.575
F(000)	2772.0
Crystal size/mm^3	0.3 × 0.28 × 0.25
Radiation	CuKα (λ = 1.54178)
2Θ range for data collection/°	6.552 to 109.012
Index ranges	-22 ≤ h ≤ 20, -20 ≤ k ≤ 19, -18 ≤ l ≤ 18
Reflections collected	19991
Independent reflections	2411 [R_{int} = 0.0670, R_{sigma} = 0.0432]
Data/restraints/parameters	2411/102/210
Goodness-of-fit on F^2	1.119
Final R indexes [I>=2σ (I)]	R_1 = 0.0891, wR_2 = 0.2974
Final R indexes [all data]	R_1 = 0.1074, wR_2 = 0.3173
Largest diff. peak/hole / e Å^{-3}	0.26/-0.29

Supplementary Table S9. Crystal pictures of oligomers **2**, **4**, **6**, **7**, **9**, **10**, and **11**.

Number	Crystal pictures
Oligomer **2**	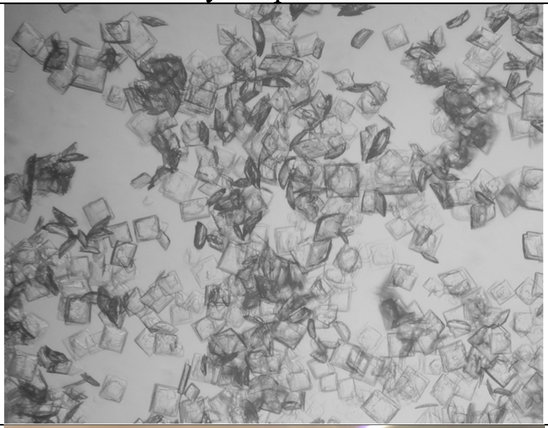
Oligomer **4**	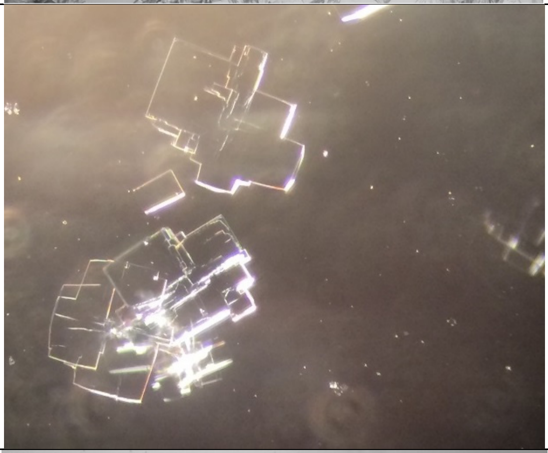
Oligomer **6**	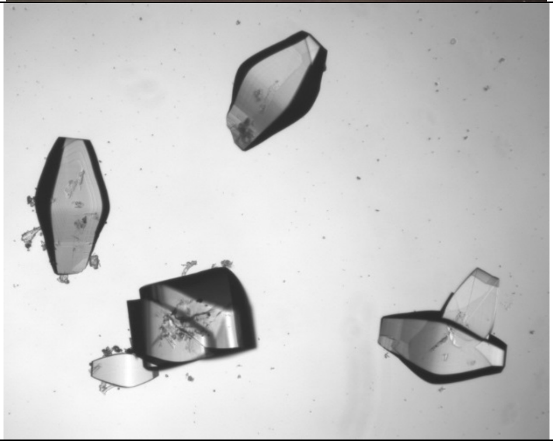

Oligomer **7**	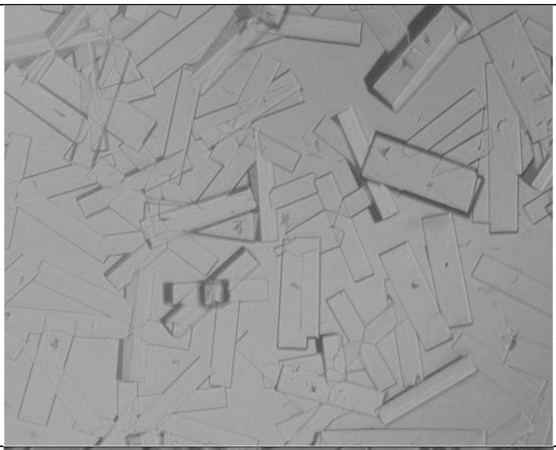
Oligomer **9**	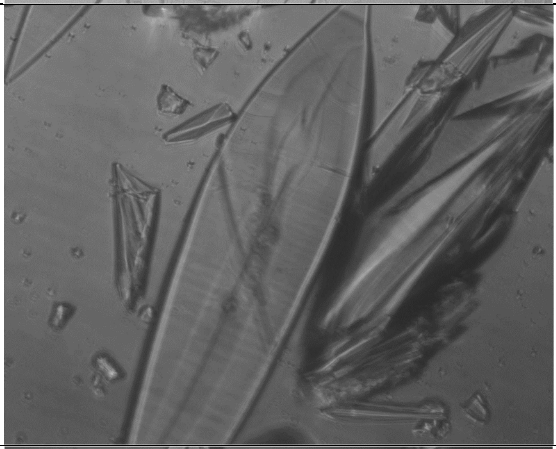
Oligomer **10**	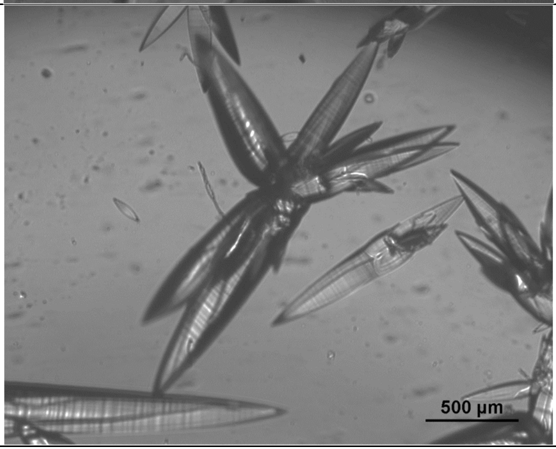

Racemate **11**	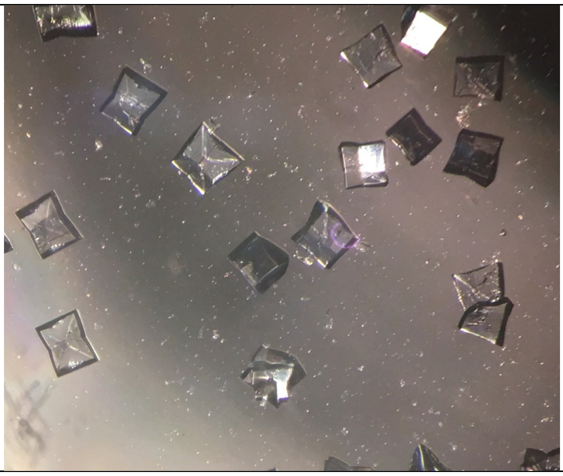

3. Supplementary References

1 Teng, P. *et al.* Hydrogen-Bonding-Driven 3D Supramolecular Assembly of Peptidomimetic Zipper. *J. Am. Chem. Soc.* **140**, 5661–5665 (2018).

2 Teng, P. *et al.* Right-Handed Helical Foldamers Consisting of De Novo d-AApeptides. *J. Am. Chem. Soc.* **139**, 7363–7369 (2017).

3 Wu, H. *et al.* New Class of Heterogeneous Helical Peptidomimetics. *Org. Lett.* **17**, 3524–3527 (2015).

4 Bruker (2016). APEX3 (Version 2015.9). Bruker AXS Inc., Madison, Wisconsin, USA.

5 Bruker (2016) SAINT V8.35A. Data Reduction Software.

6 Sheldrick, G. M. (1996). SADABS. Program for Empirical Absorption Correction. University of Gottingen, Germany.

7 Sheldrick, G.M. (1997) SHELXL-97. Program for the Refinement of Crystal.

8 Sheldrick, G. Phase annealing in SHELX-90: direct methods for larger structures. *Acta Cryst. A* **46**, 467–473 (1990).

9 Sheldrick, G. A short history of SHELX. *Acta Cryst. A* **64**, 112–122 (2008).

10 Dolomanov, O. V., Bourhis, L. J., Gildea, R. J., Howard, J. A. K. & Puschmann, H. OLEX2: a complete structure solution, refinement and analysis program. *J. Appl. Crystallogr.* **42**, 339–341 (2009).

11 Sheldrick, G. Experimental phasing with SHELXC/D/E: combining chain tracing with density modification. *Acta Cryst. D* **66**, 479–485 (2010).

12 Emsley, P., Lohkamp, B., Scott, W. G. & Cowtan, K. Features and development of Coot. *Acta Cryst. D* **66**, 486–501 (2010).

13 Spek, A. PLATON SQUEEZE: a tool for the calculation of the disordered solvent contribution to the calculated structure factors. *Acta Cryst. C* **71**, 9–18 (2015).

14 Spek, A. Structure validation in chemical crystallography. *Acta Cryst. D* **65**, 148–155 (2009).

15 Hooft, R. W. W., Straver, L. H. & Spek, A. L. Determination of absolute structure using Bayesian statistics on Bijvoet differences. *J. Appl. Crystallogr.* **41**, 96–103 (2008).
