# Supplementary material for: The folding propensity of α/sulfono-γ-AA peptidic foldamers with both left- and right-handedness
Source: Commun Chem. 2021 May 10;4:58. doi: 10.1038/s42004-021-00496-0 (PMC9814141; doi:10.1038/s42004-021-00496-0)
Supplement: Supplementary file 2 — Description of Additional Supplementary Files [file 42004_2021_496_MOESM2_ESM.pdf]

Description of Additional Supplementary Files

File Name: Supplementary Data 1

Description: CIF file for oligomer 4.

File Name: Supplementary Data 2

Description: CIF file for oligomer 6.

File Name: Supplementary Data 3

Description: CIF file for oligomer 7.

File Name: Supplementary Data 4

Description: CIF file for oligomer 7 where the solvents are masked.

File Name: Supplementary Data 5

Description: CIF file for oligomer 9.

File Name: Supplementary Data 6

Description: CIF file for oligomer 10.

File Name: Supplementary Data 7

Description: CIF file for oligomer 11.
